# Anesthetic Challenges in Managing a Patient With Traumatic Aortic Aneurysm and Dissection for Non-vascular Surgery: A Case Report

**DOI:** 10.7759/cureus.67153

**Published:** 2024-08-18

**Authors:** Aparna Bagle, Hashika Jani, Sandeep Veer, Runjhun Jain, Sharan Muruganandam

**Affiliations:** 1 Anesthesiology, Dr. D. Y. Patil Medical College, Hospital and Research Centre, Dr. D. Y. Patil Vidyapeeth (Deemed to Be University), Pune, IND

**Keywords:** anesthesia, aortic dissection, aortic aneurysm, blunt trauma chest, aortic injury

## Abstract

This case report describes the successful management of a 23-year-old male with traumatic aortic aneurysm and dissection, concomitant with bilateral lower limb fractures, highlighting the complexities and challenges of managing such a patient. The patient presented with extensive trauma, including chest pain, cough, and hoarseness of voice, and was diagnosed with a large fusiform aneurysm and dissection of the aorta. A multidisciplinary approach was adopted, and the patient underwent open reduction and internal fixation (ORIF) under combined spinal-epidural anesthesia. Meticulous hemodynamic control and vigilant monitoring ensured a stable intraoperative course. The aortic aneurysm was managed conservatively, and the patient was closely monitored for complications. This case report emphasizes the importance of interdisciplinary synergy, meticulous planning, and vigilant monitoring in managing high-risk patients and demonstrates the successful implementation of evidence-based practices in mitigating potential risks. The patient's successful outcome highlights the impact of collaboration between anesthesiologists, orthopedic surgeons, cardiovascular surgeons, and intensivists in managing complex and daunting injuries.

## Introduction

Road traffic accidents causing blunt thoracic and abdominal injuries can result in fatal aortic injuries. Although traumatic aortic injuries occur in less than 1% of motor vehicle accidents, they account for up to 33% of fatalities resulting from automobile collisions [1]. Defined as a tear in the aorta, blunt trauma aortic injury (BTAI) results from a mix of shear and stretch forces due to quick deceleration, heightened intravascular pressure, and the aorta being compressed between the front chest wall and the vertebrae [2]. Up to 80% of patients presenting with BTAI succumb on-site or before reaching the hospital [3]. Those who do survive have a high in-hospital mortality rate, or they could present asymptomatically. In such patients, it is essential to maintain a high level of suspicion and make an early clinical diagnosis to prevent catastrophic complications. An aortic aneurysm can extend, increase in size, and develop a dissection, leading to complications such as hemorrhage, aortic rupture, malperfusion, branch vessel ischemia, organ ischemia, circulatory compromise, thromboembolic events, or death [1]. The intraoperative risk of rupture of an aneurysm is contingent upon its site, dimensions, classification, severity, and symptomatology. Typically, stress and unstable hemodynamics are considered to aggravate and even lead to aneurysm rupture.

## Case presentation

**Figure 1:**
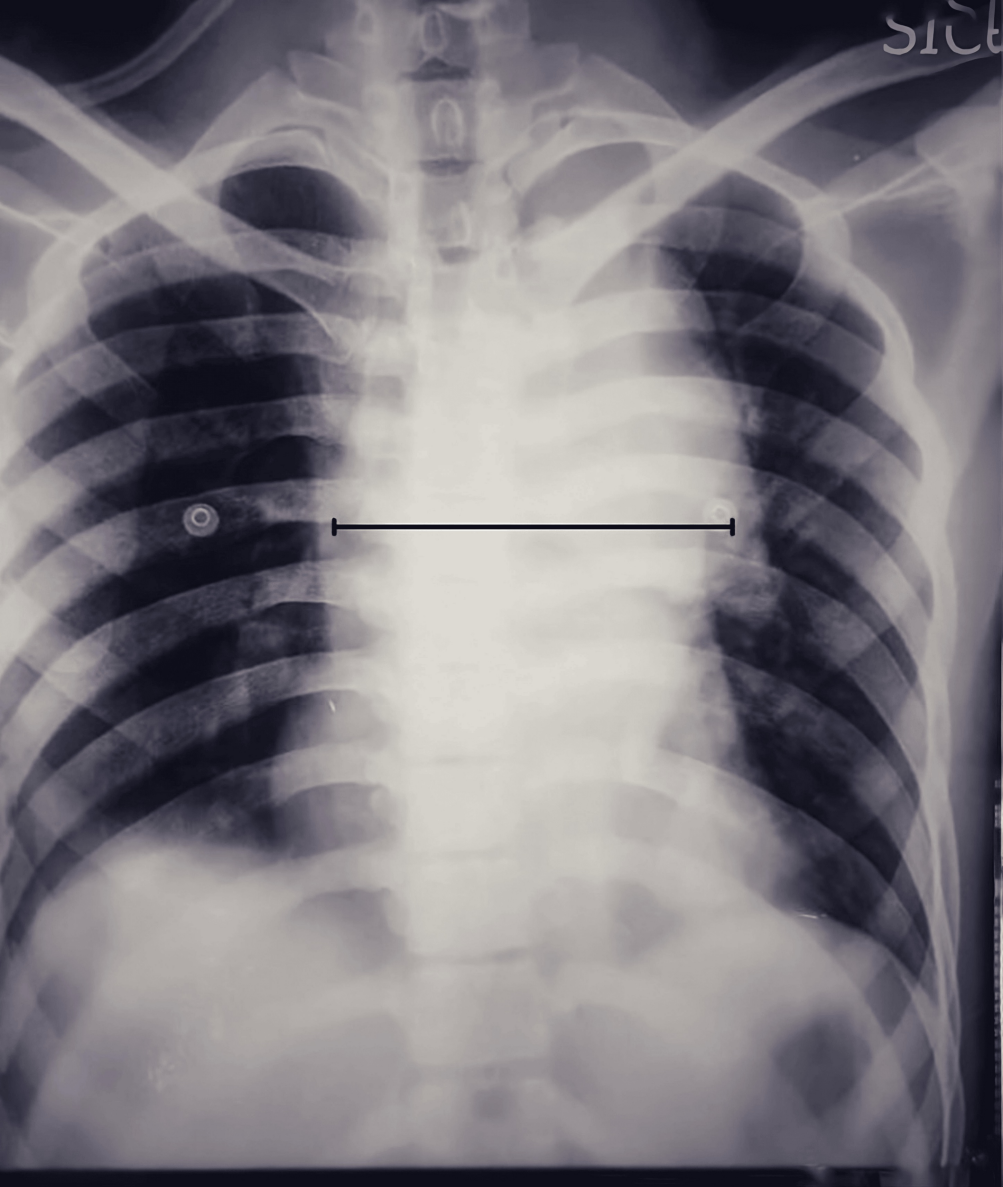
Chest radiograph, in posterior-anterior view showing widening of mediastinum due to aortic aneurysm.

We describe the case of a 23-year-old male with a history of road traffic accident, a head-on collision of his two-wheeler with a four-wheeler. The patient sustained extensive trauma to his lower limbs, abdomen, and thorax. Despite seeking medical attention at several hospitals, the patient was turned away due to the high risk and complexity of his condition. He was finally referred to our institution where a multidisciplinary team was assembled to provide comprehensive care. He presented with bilateral lower limb pain and swelling. He also complained of chest pain, cough, and hoarseness of voice. Clinical examination revealed a moderately nourished young male with feeble left radial and left dorsalis pedis artery pulse. Pulse oximetry and blood pressure were not recordable on the left arm. Chest radiography showed a widening of the mediastinum (Figure 1).

**Figure 2:**
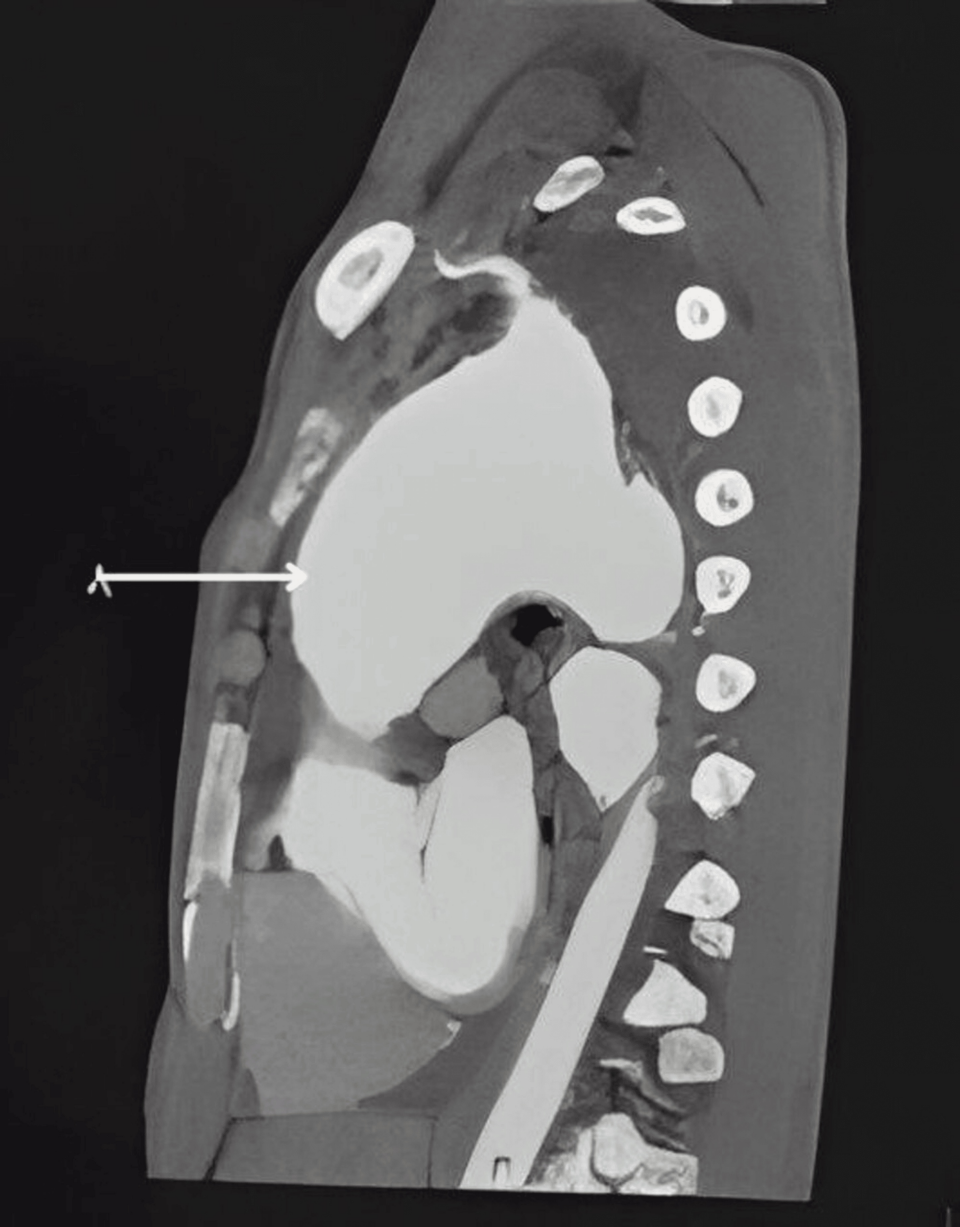
Sagittal chest computed tomography showing a large aortic aneurysm involving the ascending aorta, arch of the aorta, and proximal part of the descending aorta.

**Figure 3:**
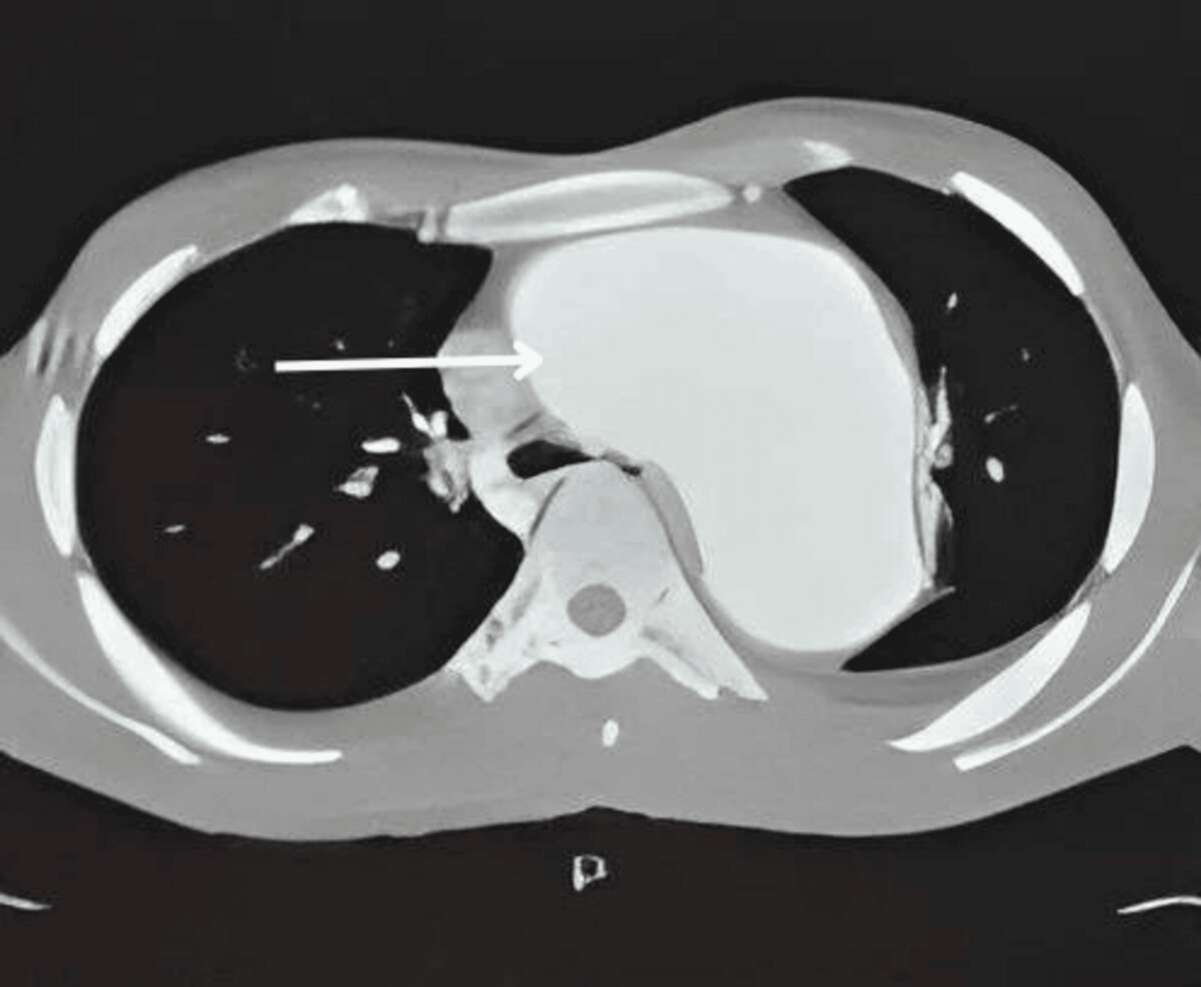
Axial chest computed tomography showing a large fusiform aortic aneurysm involving the ascending aorta, arch of the aorta, and proximal part of the descending aorta.

**Figure 4:**
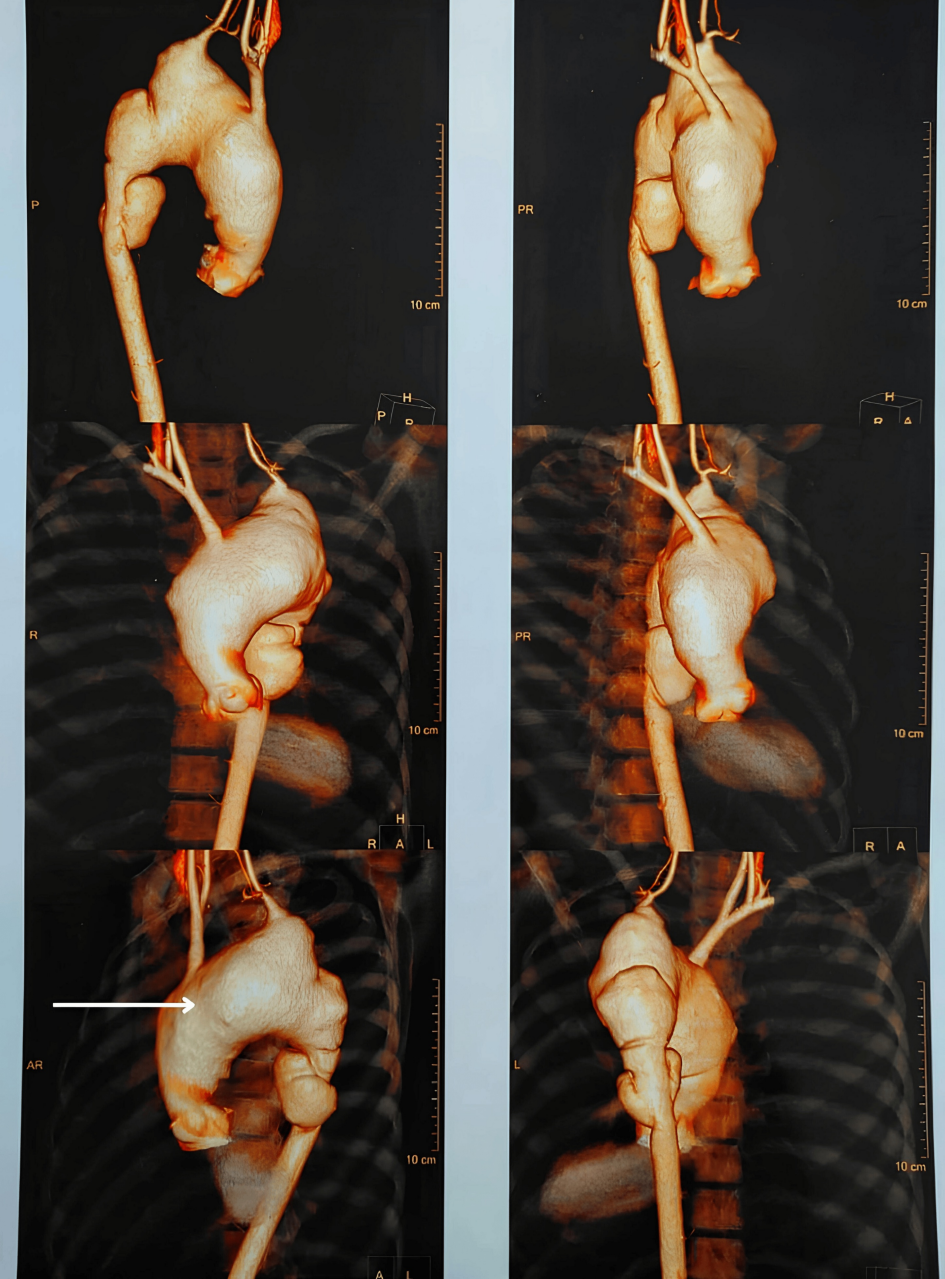
Computed tomography aortogram of the ascending aorta, arch of the aorta, and descending aorta with 3D reconstruction showing a large fusiform aortic aneurysm.

His 2D echo revealed a dilated ascending aorta and arch of the aorta, with a thin rim of pericardial effusion. Further evaluation with computed tomography (CT) thorax, pulmonary angiography, and CT aortography unveiled a large fusiform aneurysm. It rises 2 cm distal to the aortic root and extends into the arch of the aorta and proximal descending aorta. Aortic root was not involved (Figures 2-4).

**Figure 5:**
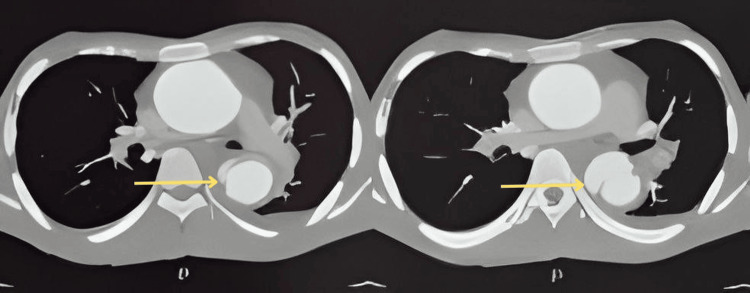
Axial chest computed tomography at the T7 and T8 level demonstrating aortic dissection in the descending aorta with an intimal flap.

A saccular aneurysm was also noted along the anterior wall in the distal thoracic aorta at the T7-T8 vertebral level with a maximum diameter measuring 38 mm. Curvilinear hypodense filling defect was noted within this aneurysm along the left lateral wall representing an intimal flap, suggestive of aortic dissection (DeBakey type lll, Stanford type B) (Figure 5).

The patient was diagnosed with a right proximal tibia fracture and a left tibia shaft fracture on bilateral lower limb radiography. After consultation with cardiovascular surgeons, it was determined that immediate surgical intervention for vascular repair was not recommended and that the aortic aneurysm would be managed conservatively and closely monitored for complications.

The patient was then planned for open reduction with internal fixation for bilateral limb fractures. Preoperatively, the patient was started on injection (inj.) of vitamin K (10 mg intramuscular once daily) (OD), tablet prednisolone (40 mg OD), and tablet metaprolol (25 mg OD). The risk arising due to anesthesia induction and surgical procedures causing hemodynamic instability and eventually fatal complications associated with aortic aneurysm were explained to the patient and his kin. On the day of surgery, postoperative intensive care unit (ICU) backup and adequate blood products were reserved. The cardiovascular thoracic surgeons and cardiac anesthesiologists were on standby with cardiac bypass should the need for emergency vascular surgery arise. Upon transferring the patient to the operation theatre all standard American Society of Anaesthesiologists (ASA) monitors were attached. Two wide-bore intravenous accesses were secured. The right radial artery was cannulated under local anesthesia for invasive blood pressure monitoring. Baseline vitals were recorded, blood pressure was measured to be 110/70 mmHg and heart rate of 76 beats per minute. The surgery was planned under combined spinal-epidural anesthesia. Under all aseptic precautions, in a sitting position, an epidural catheter was introduced in the L2-L3 intervertebral space. Then, a subarachnoid block was given with 2 ml of 0.5% inj. Bupivacaine heavy with inj. Fentanyl 25 mcg as an adjuvant at L3- L4 intervertebral space. The level of spinal blockade was fixed at T10.

Meticulous care was taken to avoid drastic fluctuations in hemodynamic parameters from baseline through close, continuous monitoring of vital signs, carefully managing blood loss, and judicious administration of vasopressor agents and intravenous fluids. This comprehensive approach ensured stable hemodynamics throughout the procedure. The surgery itself was executed without any complications, demonstrating the effectiveness of these precautionary measures. The patient was then promptly transferred to the ICU, where he received constant supervision to detect any immediate postoperative complications and ensure optimal recovery.

Currently, the patient has been placed on a rigorous follow-up regimen. These appointments are crucial during these visits, advanced imaging techniques, and clinical assessments are employed to track the aneurysm’s progression. Potential indications for vascular surgery for aneurysm repair are evaluated. This ongoing surveillance aims to pre-empt any adverse developments and to maintain the patient’s long-term health and stability.

## Discussion

This case report highlights the complexities and challenges of managing a patient with traumatic aortic aneurysm and dissection, concomitant with bilateral lower limb fractures. Such patients often present with multiple life-threatening injuries, which compete for priority. Therefore, early diagnosis, prompt intervention, and collaborative care among specialties are warranted. The anesthetic management in this case was crucial, as the patient was on the precipice of catastrophic complications. The strategic use of combined spinal-epidural anesthesia allowed us to reduce stress response, cardiovascular depression, and hemodynamic fluctuations associated with general anesthesia and ensure a stable intraoperative course.

There is literature suggesting that deliberate non-operative management of aortic aneurysm in select patients presenting with uncomplicated BTAI is a reasonable alternative [4]. Whereas for uncomplicated type B aortic dissection, medical management remains the mainstay of treatment in vitally stable patients [5,6]. Focusing on the control of systemic hypertension by employing a multimodal approach with diuretics, β-blockers, or angiotensin-converting enzyme inhibitors is pivotal. It has been demonstrated that thoracic endovascular aortic repair adds no survival advantage over medical management for uncomplicated type B aortic dissection in the short term [7]. The decision to adopt a conservative approach to managing the aortic aneurysm and dissection, upon consultation with cardiovascular surgeons, was a paradigm of individualized patient care and judicious decision-making, striking a delicate balance between the need for urgent orthopedic intervention and the risk of vascular complications. Better patient outcomes can be provided with serial imaging, a regular follow-up regimen, and vigilant surveillance, which helps to detect and prevent potential catastrophes such as aortic rupture, organ ischemia, or death, and timely interventions for the same if needed.

## Conclusions

This case report showcases the successful management of a complex case, emphasizing the importance of meticulous planning and unwavering vigilance in managing high-risk patients. The effective implementation of regional anesthesia and evidence-based practices, including goal-directed fluid management, invasive hemodynamic monitoring, and judicious vasopressor support, was crucial in mitigating the risk of devastating complications. The successful outcome demonstrates the impact of collaboration between anesthesiologists, orthopedic surgeons, cardiovascular surgeons, and intensivists, offering a ray of hope to patients facing complex and daunting injuries.

## References

[1] Harper C, Slesinger TL (2022). Traumatic aortic injuries. StatPearls [Internet].

[2] Mouawad NJ, Paulisin J, Hofmeister S, Thomas MB (2020). Blunt thoracic aortic injury - concepts and management. J Cardiothorac Surg.

[3] Teixeira PG, Inaba K, Barmparas G (2011). Blunt thoracic aortic injuries: an autopsy study. J Trauma.

[4] Caffarelli AD, Mallidi HR, Maggio PM, Spain DA, Miller DC, Mitchell RS (2010). Early outcomes of deliberate nonoperative management for blunt thoracic aortic injury in trauma. J Thorac Cardiovasc Surg.

[5] Svensson LG, Kouchoukos NT, Miller DC (2008). Expert consensus document on the treatment of descending thoracic aortic disease using endovascular stent-grafts. Ann Thorac Surg.

[6] Luebke T, Brunkwall J (2014). Type B aortic dissection: a review of prognostic factors and meta-analysis of treatment options. Aorta (Stamford).

[7] Nienaber CA, Rousseau H, Eggebrecht H (2009). Randomized comparison of strategies for type B aortic dissection: the INvestigation of STEnt Grafts in Aortic Dissection (INSTEAD) trial. Circulation.

